# A SOM-Based Membrane Optimization Algorithm for Community Detection

**DOI:** 10.3390/e21050533

**Published:** 2019-05-25

**Authors:** Chuang Liu, Yingkui Du, Jiahao Lei

**Affiliations:** School of Information Engineering, Shenyang University, Liaoning 110044, China

**Keywords:** community detection, membrane algorithm, self-organizing map network, complex networks, optimization

## Abstract

The real world is full of rich and valuable complex networks. Community structure is an important feature in complex networks, which makes possible the discovery of some structure or hidden related information for an in-depth study of complex network structures and functional characteristics. Aimed at community detection in complex networks, this paper proposed a membrane algorithm based on a self-organizing map (SOM) network. Firstly, community detection was transformed as discrete optimization problems by selecting the optimization function. Secondly, three elements of the membrane algorithm, objects, reaction rules, and membrane structure were designed to analyze the properties and characteristics of the community structure. Thirdly, a SOM was employed to determine the number of membranes by learning and mining the structure of the current objects in the decision space, which is beneficial to guiding the local and global search of the proposed algorithm by constructing the neighborhood relationship. Finally, the simulation experiment was carried out on both synthetic benchmark networks and four real-world networks. The experiment proved that the proposed algorithm had higher accuracy, stability, and execution efficiency, compared with the results of other experimental algorithms.

## 1. Introduction

Many networks can be simulated by complex networks, such as social networks, biological networks, and the World Wide Web. The study of complex networks is increasingly attracting the attention of researchers from many different fields. These complex networks are represented by nodes and edges. In order to clearly understand the structural characteristics and functional characteristics of complex networks, finding the relationship between these nodes and edges is especially important for studying the composition of the network and understanding the functional characteristics of the network. As a method to revealing the relationship between nodes and edges in the network, community structure has become a hot research topic in network science. More and more researchers are paying attention to community detection problems in complex networks [1,2,3].

There are many algorithms for studying community detection, including the graph partitioning algorithm, hierarchical clustering, modularity optimization algorithm, label propagation algorithm, partition-based clustering algorithm, evolutionary algorithm, etc. [4]. Among many algorithms, evolutionary algorithms can solve the problems of community detection without prior knowledge. These problems need to be converted into optimization problems first, and then they can be solved by using evolutionary algorithms, such as the genetic algorithm (GA), particle swarm optimization (PSO), differential evolution (DE), etc. Such algorithms have the ability to automatically detect the number of communities when the number of communities in the network is unknown, and is more suitable for solving real network problems [1]. The application of evolutionary algorithms in complex networks has attracted the attention of many researchers. Tasgin et al. first proposed a genetic algorithm to solve these kinds of complex problems [5]. Pizzuti proposed a GA-based community detection algorithm, which introduced a genetic representation and the concept of community score as the fitness function to detect community structure in complex networks [6]. Pizzuti proposed a multiobjective genetic algorithm to find communities in complex networks. The method maximizes the intra-connections inside each community and minimizes inter-connections between different communities [7]. Gong et al. proposed a synergy of a genetic algorithm with a hill-climbing strategy as the local search procedure to optimize modularity destiny to explore the network at different resolutions [8]. Pizzuti et al. proposed a many-objective optimization algorithm for community detection in multi-layer networks [9]. Meo et al. proposed a scalable method to maximize modularity in large networks, which is a new clustering method that couples the accuracy of global approaches with the scalability of local methods [10]. Grass-Boada et al. proposed a multi-objective overlapping community detection algorithm, which is based on the Pareto-dominance based multi-objective evolutionary algorithmsand global and local approaches for discovering overlapping communities [11]. Berahmand et al. proposed a local approach by detecting and expanding core nodes through extended local similarity of nodes [12]. Shi et al. proposed a locally-biased spectral approximation approach to adapt the Lanczos method for local community detection, which apply a fast random walk, personalized PageRank, and heat kernel diffusion [13]. Moradi et al. proposed an extension genetic algorithm with a novel local search strategy for community detection [14].

In summary, the research results of community detection based on evolutionary algorithms mainly focus on network coding, group initialization, evolution rule design, and objective function selection. Although the above literature has obtained a wealth of research results, the accuracy and complexity of these algorithm still needs to be improved. In this paper, we proposed an evolutionary membrane community detection algorithm based on self-organizing maps (SOM), named EMCD-SOM. SOM, an unsupervised learning algorithm for clustering and high-dimensional visualization, is an artificial neural network developed by simulating the characteristics of the human brain’s processing signals [15]. The proposed algorithm consists of objects, reaction rules, and membrane structure. An object presents a partition result of the complex network. Reaction rules include GA and DE. In the skin membrane, GA is utilized as reaction rules to evolve the objects. DE is introduced as reaction rules in the region of each membrane. In order to find the optimal membrane structure, SOM determines the number of membranes by learning the information of the objects. To evaluate the performance of EMCD-SOM, synthetic benchmark networks and four real-world networks were conducted by the proposed EMCD-SOM. The experimental results showed that the proposed method was more useful and effective than other state-of-the-art algorithms including FastNewman [16], LconDanon [17], GA-NET [6], CMM [18], and Meme-net [8] from the literature.

The main contributions of this paper are summarized below:

- The SOM neural network may learn and mine the structure of the current objects in the decision space, which is beneficial for guiding the local and global search of the proposed algorithm;
- The number of membranes of the proposed EMCD-SOM is determined according to the characteristics of SOM mapping similar data to adjacent neurons.
- GA and DE are employed as reaction rules to evolve the objects in the different region of membrane;
- The proposed EMCD-SOM can implement the balance of exploration and exploitation in four real world networks.

The rest of this paper is organized as follows. In Section 2, the description of the proposed EMCD-SOM is elaborated. In Section 3, the simulation results are evaluated on the benchmark test problems in comparison with some state-of-the-art evolutionary algorithms. Moreover, this section includes a sensitivity analysis for the proposed EMCD-SOM. Finally, Section 4 summarizes the concluding remarks of this paper.

## 2. The Proposed Approach

This section will explain the principles of the proposed EMCD-SOM based on a membrane system. Since the membrane system consists of three elements: object, reaction rule, and membrane structure, the proposed algorithm also has these elements. In the proposed EMCD-SOM, the focus is on how to achieve these three elements. The object as the first element in the region of membrane represents candidate solution for network partitioning. The second element is the reaction rule, which are designed to evolve objects in different region of membranes. The membrane structure is the last element, which helps to promote the exchange of information between membranes and enhance the diversity of objects. These features are very useful in developing a new evolutionary algorithm to improve its solving performance.

The pseudo-code of the proposed EMCD-SOM is given in the Algorithm 1.

**Algorithm 1** The pseudo-code of the proposed EMCD-SOM.
**Input:** The parameters of the proposed algorithm are initialized, including the number of objects in each elementary membrane, each object within its boundaries.

**Output:** The best object is found from the different elementary membranes.

 1: The objects are initialized from the search space.

 2: The fitness of these objects is calculated according to the modularity density function in Equation (3).

 3: **while** End Condition **do**

 4:    Determining the number of membrane (*NC*) by invoking SOM

 5:    **for** *i* = 1; *i* < *NC*; *i*++ **do**

 6:        Evolving the objects in the region of elementary membrane according to the DE-based reaction rule.

 7:    **end for**

 8:    The objects from the region of elementary membrane are released into the region of skin membrane.

 9:    All objects in the region of skin membrane are evolved according to the GA-based reaction rule.

10:** end while**


### 2.1. Object and Its Initialization

**Figure 1:**
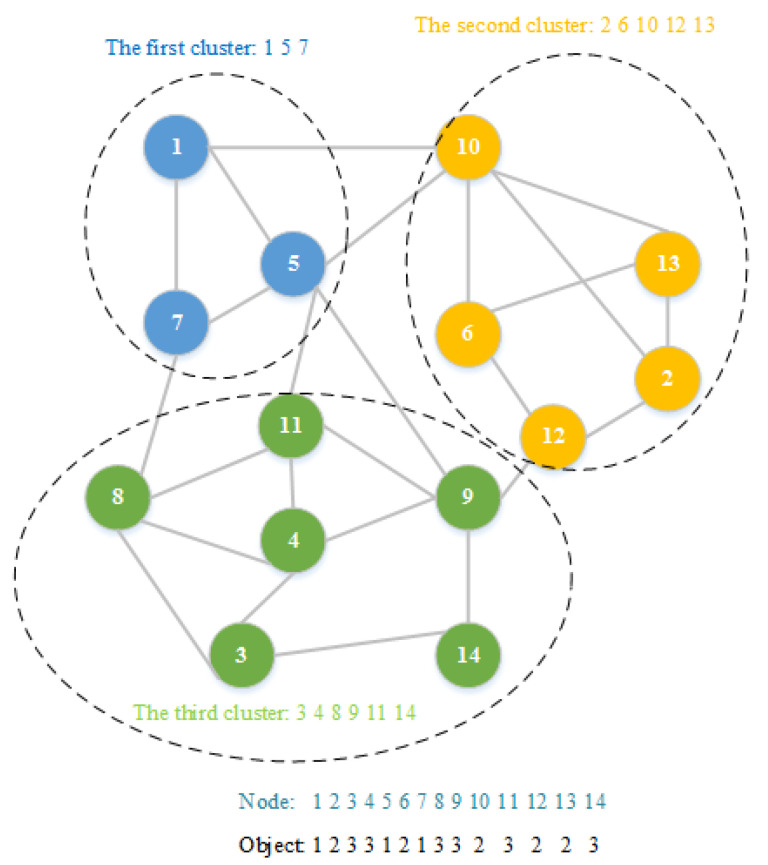
A generic illustration of the representation of a discrete object.

The object is encoded as a partition of community in the complex network. Depending on the number of network communities, each object can be represented as a set of real integer values. In the proposed algorithm, an object is defined as:

(1) $$X=(x_{1},x_{2},\cdots ,x_{n})$$

where *n* represents the number of the nodes in a complex network, and *x_i_* is the *i*-th node and is an integer change from 1 to *n*. A community consists of nodes with the same value. The graphical illustration of the object coding is shown in Figure 1. As can be seen from Figure 1, there are 14 nodes and a total of three communities represented by objects. It is worth mentioning that the number of communities is automatically determined by the proposed algorithm. In the worst case, a complex network with *n* nodes can be divided into *n* communities.

The object represents the result of network partitioning in the proposed EMCD-SOM. It is initialized according to Equation (2):

(2) $$x_{i,j}=\left\lceil x_{j}^{l}+\left(x_{j}^{u}-x_{j}^{l}\right)\times r\right\rceil +1$$

where 1 ≤ *i* ≤ *N*, *N* is the number of objects in the region of all membranes. 1 ≤ *j* ≤ *n*, *n* represents the maximum value of the node identifier in a complex network. *x_i,j_* is the value of the *j*-th identifier in the *i*-th object, which is an integer value from 1 to *n*. $x_{j}^{l}$ represents the *j*-th lower limit of the identifier in the complex network, which has a value of 1, and $x_{j}^{u}$ represents the upper boundary value of the *j*-th identifier of the identifier in the complex network, which is *n*. *r* can generate a random number on the interval (0, 1). In the formula, the ceiling operations is utilized to ensure that *x_i,j_* is an integer value.

### 2.2. Objective Function

Among many objects in the region of membranes, how to determine which object is the best forthe best community partition requires the use of the objective function. The modularity density widely used in community detection problems [19], and its definition is given in Equation (3).

(3) $$f=\sum_{i=1}^{N}\left(\frac{L\left(V_{i},V_{i}\right)-L\left(V_{i},\bar{V_{i}}\right)}{|V_{i}|}\right)$$

where $L\left(V_{1},V_{2}\right)=\sum_{i\in V_{1},j\in V_{2}}A_{ij}$, and $L\left(V_{1},\bar{V_{2}}\right)=\sum_{i\in V_{1},j\in \bar{V_{2}}}A_{ij}$, and $\bar{V_{2}}=\Omega -V_{2}$, and *A* is the adjacent matrix of the network, and $\Omega =V_{1},V_{2},\cdots ,V_{N}$ is a partition.

The value of the objective function is one of the most critical steps that guides the object’s search direction. The modularity density values are utilized to evaluate the quality of objects in all membranes. The higher modularity density value has, the better community structure is attained by the proposed algorithm. If the modularity density value is equal to 1, the network partition represents a very good community structure.

### 2.3. Membrane Structure

Since the proposed algorithm is based on a membrane system, it inherits the same network structure from the membrane system. In order to simplify the implementation of this structure, the proposed algorithm is defined as a structure containing only the elementary membrane. Each elementary membrane can be thought of as an evolutionary unit. In the experiment, we found that the number of membranes is difficult to set. To solve this problem, we used a self-organizing mapping network (SOM) to determine the number of elementary membranes, specifically using SOM to discover the structural information of the decision space of objects, and then determine the number of elementary membranes. The details of SOM are given below.

**Figure 2:**
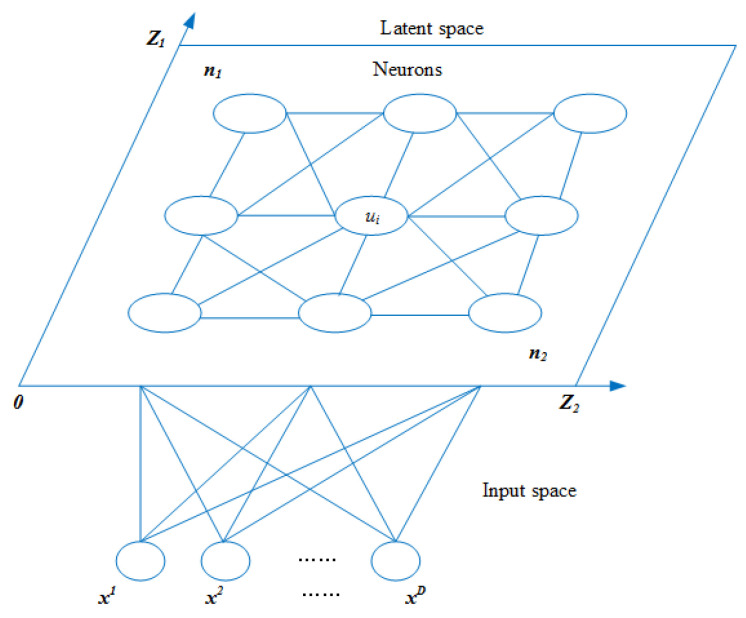
An illustration of a two-dimensional self-organizing map network (SOM).

SOM, an unsupervised learning algorithm proposed by Kohonen for clustering and high-dimensional visualization, is an artificial neural network developed by simulating the characteristics of the human brain’s processing signals. It is characterized by the ability to map high-dimensional distributions to low dimensions and maintain mapping invariance. In recent years, SOM have been applied to the solution of optimization problems. Jin et al. proposed a SOM with a novel learning rule to solve the traveling salesman problem (TSP) [20]. Villmann et al. proposed a hybrid system combining SOM and evolutionary algorithms to promote neighborhood cooperation [21]. Zhang et al. proposed a self-organizing multiobjective evolutionary algorithm. SOM is employed to establish the neighborhood relationship among current solutions [22]. Liang et al. proposed a multi-objective particle swarm optimization algorithm based on SOM, which mainly uses SOM to discover the structural information of population and the multi-objective Pareto solution set, and then guides the particle flight [23]. The topology of a two-dimensional SOM is shown in Figure 2.

As shown in the figure, SOM consists of an input layer and a competition layer (output layer). The number of input layer neurons is *D*, and the competition layer consists of a one-dimensional or two-dimensional planar array of $N=n_{1}\times n_{2}$ neurons. Each neuron $u_{i}\in \left(1,2,\cdots ,N\right)$ has its own location information $Z^{u_{i}}=\left(z_{1}^{u_{i}},z_{2}^{u_{i}}\right)$ and weight information $W^{u_{i}}=\left(w_{1}^{u_{i}},w_{1}^{u_{i}},\cdots ,w_{D}^{u_{i}}\right)$. The network is fully connected, that is, each input node is connected to all output nodes. SOM consists of a training phase and a clustering phase. In the first stage, the training data is randomly selected, the winning neurons are selected according to the Euclidean distance, and the weights of the winning neurons and their neighboring neurons are updated. The second stage is mapping test data to neurons and similar data to neighboring neurons. The number of membranes of the proposed EMCD-SOM is determined according to the characteristics of SOM mapping similar data to adjacent neurons. Furthermore, the number of clusters in the SOM is used to determine the number of membranes in the proposed algorithm. The structure of EMCD-SOM is conducive to improving search efficiency and is suitable for solving community detection problems.

In the proposed algorithm, the objects in the region of elementary membrane are evolved by the reaction rule according to the differential evolution algorithm. When objects from different membranes are evolved, they are released into the region of the skin membrane. These objects will continue to evolve by calling genetic algorithm-based reaction rules. Then, they are aggregated into several classes using SOM and these clustered objects are in turn sent to the region of elementary membrane and are evolved by invoking the reaction rule. After executing several generations, some good objects can be generated by executing reaction rules in the different elementary membranes. The best object can be found by comparing the modularity density values of these objects.

### 2.4. Reaction Rules

The reaction rule is inspired by the chemical reaction of the objects and the way of handling the compound. Reaction rules can be implemented through mechanisms that can develop objects into the direction of the global optimal partition of the network. According to “No Free Lunch”, there is no single optimization algorithm to solve every optimization problem effectively and efficiently. In other words, different algorithms possess a different accuracy to solve the same optimization problem. The ensemble of state-of-the-art algorithms can obtain a better solution than using a single algorithm. Inspired by this, we employed the GA algorithm and the DE algorithm to evolve objects in both the skin membrane and elementary membrane.

GA is a computational model that simulates the natural evolution of Darwin’s biological evolution theory and the biological evolution process of genetic mechanism. It is a method to search for optimal solutions by simulating natural evolutionary processes. In each generation, the optimal individual is selected based on the individual’s adaptability in the problem domain, and new individuals are generated by crossover and mutation operations in the genetic operator. In the proposed algorithm, GA acts as a reaction rule in the skin membrane. More specifically, the individual in GA is represented by the object. The selection operation is used to select the parent population of mating in the GA. Here we used a wide range of deterministic tournament selection operators. The crossover operation was implemented by two-way crossing over operation in the literature [8]. In mutation, we randomly selected a object in the region of the skin membrane. A point mutation was employed, which randomly picked a dimension value on the object and then randomly changed the value to its neighbor’s dimension value. GA facilitated global search by the proposed algorithm. The parameters of GA were given as follows: Crossover probability = 0.8, mutation probability = 0.2.

DE was employed as a reaction rule in elementary membranes. DE is an optimization algorithm based on differential and simple mutation operation and one-to-one competitive survival strategy, which reduces the complexity of genetic operations. It generates new individuals through differential mutation with some different strategies including DE/rand/1, DE/best/1, DE/best/2, DE/rand-to-best/1, etc. In order to improve the diversity of candidate solutions, DE introduces crossover to operate on target vectors and mutation vectors to generate new experimental vectors. In the proposed algorithm, DE/best/1 was utilized to evolve objects in the region of the elementary membrane. A modified binomial crossover was employed to assign the value of either dimension in an object to the value of the corresponding dimension in another object [24]. The parameters of DE were given as follows: *F* = 0.9 is called the differential weight. *CR* = 0.3 is called the crossover probability.

## 3. Experimental Evaluation

The performance of the proposed algorithm was validated in a series of experiments based on both synthetic benchmark networks and the four real-world networks by comparing it with state-of-art algorithms. Section 3.1 will discuss the details of these networks. Section 3.2 will describe the experimental condition in running the simulation. Section 3.3 will give several metrics of the experimental algorithms. Section 3.4 will give the simulation result of the LFR (Lancichinetti–Fortunato–Radicchi) benchmark network calculated by all experimental algorithms. Section 3.5 will discuss the experimental results based on the evaluation metrics of the experimental algorithm on different network datasets.

### 3.1. Synthetic Benchmark Networks and Four Real-World Networks

#### 3.1.1. Description of Synthetic Benchmark Betworks

The first set of experiments is the LFR benchmark network presented by Lancichinetti and Radicchi in [25], which has power law degree distribution and variable sized communities. It is the most widely used benchmark network for testing the performance of algorithms in community detection. Compared with other synthetic networks, LFR networks can reflect some important features of complex real-world systems. In the simulation, the number of nodes in the LFR network was 1000, the average degree was 15, the maximum degree was 50, the mixing parameter was 0.1, the minimum planted community size was 20, and the maximum planted community size was 50.

#### 3.1.2. Description of Four Real-World Networks

In the following experiments, four real-world networks were employed to test the performance of the proposed algorithm, including the Zachary’s karate club network, American college football club network, Krebs America Political Book network, and Bottlenose dolphins network. The ground-truths of these networks has been known. More details about the definition of these network datasets can be discussed as follows. The Zachary’s karate club network, constructed by Zachary, is a network of relations between 34 members of a karate club over a period of two years [26]. The karate club is split into two communities of almost the same size on account of disagreements between the administrator and the instructor of the club. The American college football network consists of 115 vertices and 613 edges, which is divided into 12 communities, which was first proposed by Girvan and Newman [27]. Vertices in the network represent teams which are identified by their college names, and edges represent the regular season games between the two teams they connect. This Krebs America political book network consists of 105 vertices and 441 edges between books purchased together during the 2004 presidential election, which was compiled by Krebs [28]. Bottlenose Dolphins network consists of 62 vertices and 60 edges based on social acquaintances, which is naturally divided into two large groups: The male group and the female one [29]. Each node represents a dolphin living over a period of 7 years in the bottlenose dolphins network. The related parameters of each real-world network are described in Table 1.

### 3.2. Experimental Conditions

In the experiments, some related community detection algorithms were employed to compare with the proposed algorithm. These algorithms consist of Fast–Newman, Lcon-Danon, GA-net, Meme-net, and MOGA-net. Some of them, including GA-net and Meme-net, are single-objective algorithms, while the rest are non-evolutionary algorithms. They were run in Windows 7 enterprise version under the hardware environment of Intel Pentium dual-core 2.93 GHZ and 16 GB RAM. The proposed algorithm was implemented using Matlab2015.

Since the results of the community detection method based on evolutionary algorithm depend on the validity of the random search process, 30 repeated tests were performed independently on both synthetic benchmark networks and 4 real-world networks, and statistical results were calculated in order to evaluate the statistical performance of algorithms and reduce statistical errors. Moreover, 4 statistical metrics were designed, such as Mean, Std, Worst, and Best. These metrics were employed to evaluate the solving performance of these various algorithms.

### 3.3. Evaluation Measures

At present, there are many metrics for evaluating the effectiveness of community detection algorithms that detect the quality of network partitions of complex networks. Among these metrics, the normalized mutual information (NMI) are the most widely used in community detection of complex networks. In addition, to further evaluate the quality of the experimental results, some clustering indicators were introduced include the F-measure and Rand Index.

NMI is a similarity measure estimating the similarity between detected partitions and true ones. A higher NMI value represents a greater similarity between two partitions. If NMI takes its maximum value which is equal to 1, all communities obtained by the experimental algorithms are identical to all real communities. In the following experiment, NMI was used to evaluate the results between true partition and the partition obtained by experimental algorithms. The definition of NMI(A, B) is shown in Equation (4):

(4) $$NMI\left(A,B\right)=\frac{-2\sum_{i=1}^{C_{A}}\sum_{j=1}^{C_{B}}D_{ij}\log\left(\frac{D_{ij}N}{D_{i}\cdot D_{j}}\right)}{\sum_{i=1}^{C_{A}}D_{i}\cdot \log\left(\frac{D_{i}}{N}\right)+\sum_{j=1}^{C_{B}}D_{j}\cdot \log\left(\frac{D_{j}}{N}\right)}$$

where *A* and *B* are partitions of a network, and *C_A_* represents the number of communities in *A* while *C_B_* denotes that of *B*. *D* is a confusion matrix, and *D_i,j_* stands for the number of nodes in community *i* of *A* that also appear in community *j* of *B*. *N* is the number of elements. *D_i_* is the sum over row *i* of *D* while *D_j_* is the sum of elements in column *j*.

F-measure is also called F-score, which is a weighted harmonic averaging of Precision and Recall. It is a commonly used evaluation standard in the clustering field and is often used to evaluate the quality of the classification model. The definition of F-measure is shown in Equation (5):

(5) $$F=2\times \frac{PR}{P+R}$$

where *P* is the precision and *R* is the recall rate.

Rand Index (RI) is also called Rand measure, which is a measure of the similarity between two data clusterings. In the experiments, Rand Index is employed to measure the similarity between real partitions and the partitions obtained by experimental algorithms. The definition of Rand Index is shown in Equation (6):

(6) $$RI=\frac{a}{a+b}$$

where *a* can be considered as the number of agreements between real partitions and the partitions obtained by experimental algorithms, and *b* as the number of disagreements between real partitions and the partitions obtained by experimental algorithms.

### 3.4. Experiments on Synthetic Benchmark Networks

In the following experiment, the LFR network consisted of a network of size 1000 with a mixing parameter fixed at 0.1. All experimental algorithms ran independently 30 times in the networks. The statistical results of the evaluation indicators with NMI, F-measure, and Rand Index were used to evaluate the performance of all experimental algorithms.

As shown in Table 2, the proposed EMCD-SOM achieved the best results on all indicators in comparison with other experimental algorithms. FastNewman had suboptimal results on the synthetic benchmark networks. Due to the fact that Meme-net runs for a long time and there is no calculation result, the statistical result was represented by ‘-’. In summary, compared with other experimental methods, the proposed algorithm was suitable for solving networks with a large number of nodes.

### 3.5. Experiments on Real-World Networks

In this section, the proposed algorithms were compared with other algorithms for 4 real-world datasets with real partitions known in the following experiment. All experimental algorithms were run 30 times, independently. The statistical results of NMI, F-measure, and Rand Index were utilized to evaluate the performance of the experimental algorithms.

#### 3.5.1. Display Network Partition

**Figure 3:**
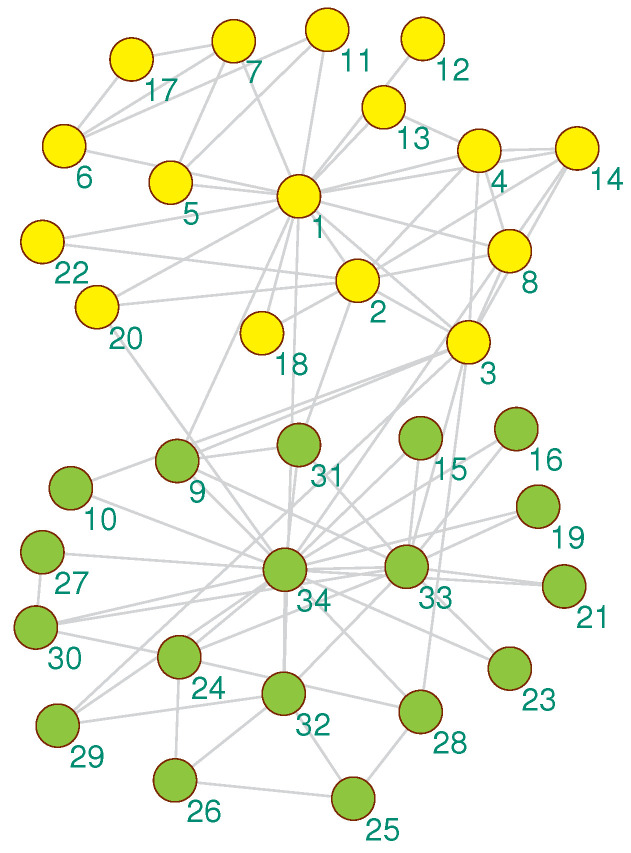
The community detection result of the proposed algorithm on Zachary’s karate club network.

**Figure 4:**
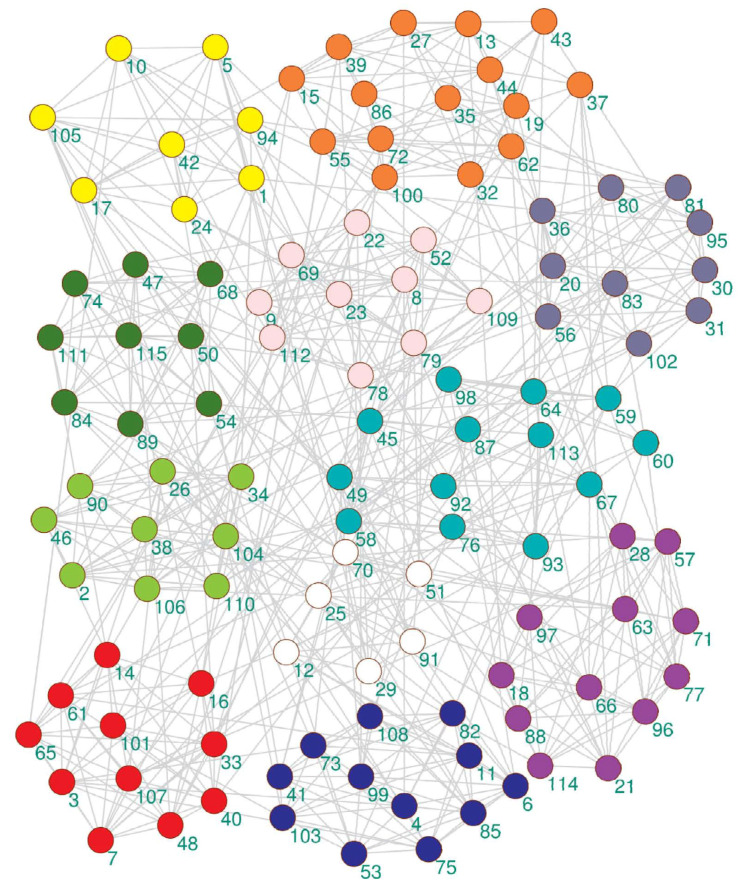
The community detection result of the proposed algorithm on the American college football club network.

**Figure 5:**
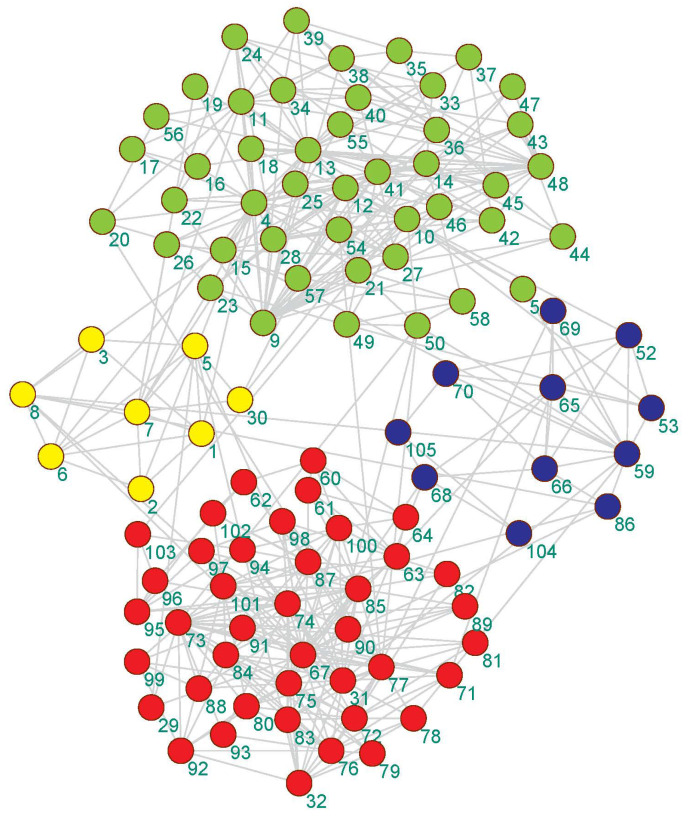
The community detection result of the proposed algorithm on the Krebs America political book network.

**Figure 6:**
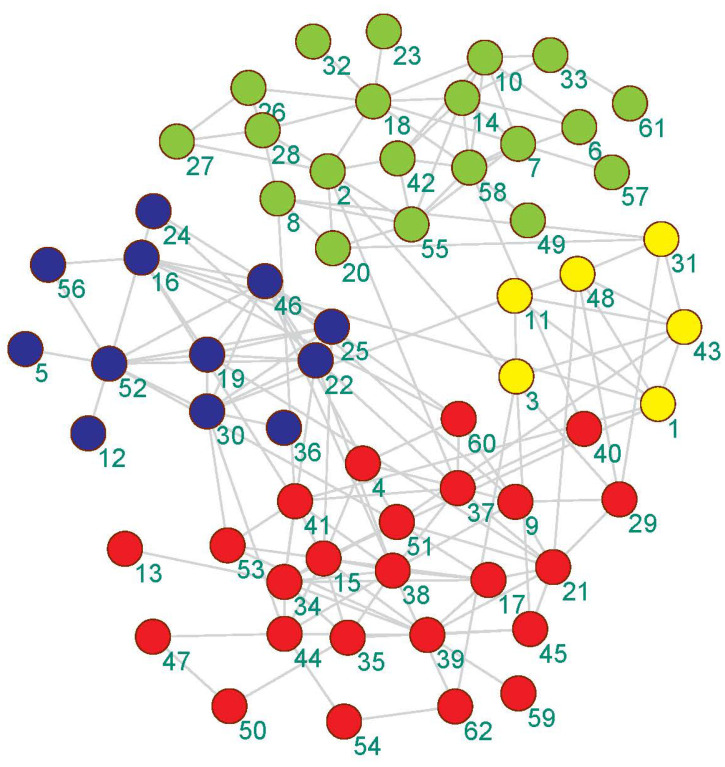
The community detection result of the proposed algorithm on the Bottlenose dolphins network.

We visualized the community detection results obtained by the proposed algorithm on 4 real-world datasets with real partitions known. As shown in Figure 3, Figure 4, Figure 5 and Figure 6, the community division was the best result from 30 runs, and almost every partition had a good community structure and was similar to the real division of the network. The results of Figure 3 show that the proposed algorithm can obtain different levels of community structure on Zachary’s karate club network. The proposed algorithm could discover 2 communities, as shown in Figure 3, which is consistent with the real community structure in Table 1.

The community structure detected by the proposed algorithm on the American college football network is shown in Figure 4. It can be seen from Figure 4 that the proposed algorithm detected 11 partitions, but only a few nodes had community partitioning errors. The real network had 12 partitions in Table 1.

As seen Figure 5 in the US political book network, due to the complexity of the network structure, the proposed algorithm had a community structure with 4 communities, but the actual network partition was 3 in Table 1.

Lastly, Figure 6 shows the results of the community of the Bottlenose dolphins network obtained by the proposed algorithm. As shown in Figure 6, the number of the community obtained by the proposed algorithm was larger than the result of the real network in Table 1.

#### 3.5.2. Comparison of the Proposed Algorithm with Other Algorithms

In this section, Table 3, Table 4 and Table 5 show the community detection effect of the proposed algorithm and other experimental algorithms running 30 times with 3 evaluation indicators on 4 real networks. As shown in Table 3, Table 4 and Table 5, compared to other algorithms, the proposed algorithm had a good performance in community detection on 4 real-world networks.

The NMI values of all experimental algorithms are shown in Table 3. On Zachary’s karate club network, the best results obtained by the proposed algorithm indicated that it can all converge to the global optimal *NMI* = 1. The result indicates that the community obtained by the proposed algorithm was exactly the same as the real community. This result can also be obtained from Figure 3. To illustrate the performance of the proposed algorithm, we sorted these algorithms according to the average of the NMI indicator as follows: CMM, Meme-net, EMCD-SOM, FastNewman, GA-NET, and LconDanon. Compared with Meme-net, the proposed algorithm obtained the suboptimal community partition result.

On the American college football club network, the proposed algorithm gained the best average NMI of 0.900987 in all experimental algorithms. CMM attained the second-best NMI average. The performance of these algorithms was sorted as follows: EMCD-SOM, CMM, Meme-net, LconDanon, FastNewman, and GA-NET.

On Krebs America political book network, the proposed algorithm found the second-best NMI average of 0.528597, which is not much different from FastNewman. The best result, out of the 30 times, belonged to the proposed EMCD-SOM. According to the average value of NMI, these algorithms were sorted as follows: FastNewman, EMCD-SOM, LconDanon, Meme-net, CMM, and GA-NET.

On the Bottlenose dolphins network, the proposed algorithm obtained the fourth average. These algorithms were sorted as follows: CMM, LconDanon, FastNewman, EMCD-SOM, Meme-net, and GA-NET.

Next, all experimental algorithms were evaluated by calculating the F-measure, which was conducted on the real-world networks. This indicator is often used to evaluate the quality of the classification model. The F-measure values obtained by the experimental algorithms on real-world networks are shown in Table 4.

As seen in Table 4, the proposed algorithm could obtain the best results for the F-measure indicator compared with all experimental algorithms on most of real-world networks. Compared with the proposed algorithm, CMM gained the best result on Dolphins, and Meme-net gained the best result on Karate Club, and FastNewman gained the best result on Political Book and Dolphins.

Finally, all experimental algorithms were evaluated according to the Rand Index indicator. This indicator is often used to measure the similarity between two data clusterings. The Rand Index values obtained by the experimental algorithms on the real-world networks are shown in Table 5.

As we can see, compared with the other 5 community detection methods for Rand Index on real networks, the proposed EMCD-SOM could get satisfactory results, especially in the American college football club network. For the karate network, Meme-net gained the best result. For Football club, the proposed algorithm gained the best result. FastNewman gained the best result on the Political book and Dolphins network in terms of the Rand Index. It is worth noting that the proposed algorithm was similar with FastNewman on the Political book network.

Finally, although the proposed algorithm was not optimal, the proposed algorithm showed stable results on different networks, which indicates that the proposed algorithm is suitable for solving community structure partitioning problems in complex networks.

## 4. Conclusions

This paper proposed a membrane algorithm based on a self-organizing map network named EMCD-SOM, which was used to solve complex network community detection problems. According to the characteristics of community detection, the proposed algorithm gave the realization principle of object, reaction rule, and membrane structure. The encoded object represented the partitioning result of community detection. Genetic algorithm and differential evolution were employed as two reaction rules to evolve objects in different regions of the membranes. The proposed algorithm used SOM to determine the number of elementary membranes and fully exploit neighborhood information. The effectiveness of the proposed algorithm was evaluated on four real-world networks. Compared with other algorithms, the results showed that our algorithm could achieve better performance, indicating that EMCD-SOM has great potential in solving community detection problems. In addition, because EMCD-SOM adopts modularity density as an objective function, it can effectively solve the resolution limitation problem of the modularity degree, and reasonably divide the network structure at different resolutions. In the future, EMCD-SOM will be improved so that it can effectively detect communities in overlapping networks, large-scale networks, and multi-level heterogeneous networks.

## Figures and Tables

**Table 1:** Parameters of the real-world networks.

Datasets	Nodes	Edges	Communities
Zachary’s karate club network	34	78	2
American college football club network	115	613	12
Krebs America political book network	105	441	3
Bottlenose dolphins network	62	60	2

**Table 2:** The statistical values obtained by the experimental algorithms on the synthetic benchmark networks of size 1000 with a mixing parameter fixed at 0.1. GA-NET: GeneticAlgorithm-NET; CMM: Convexified Modularity Maximization; Meme-net: Memeticalgorithm-net; EMCD-SOM: The proposed algorithm; NMI: normalized mutual information; RI: Rand Index.

Metrics	Statistics	FastNewman [16]	LconDanon [17]	GA-NET [6]	CMM [18]	Meme-Net [8]	EMCD-SOM
NMI	Mean	0.952684	0.945996	0.872757	0.939711	-	0.992237
	Std	5.64601 × 10^−16^	0	0.0186498	0.0136735	-	0.0115922
	Worst	0.952684	0.945996	0.827308	0.915167	-	0.947601
	Best	0.952684	0.945996	0.899495	0.969452	-	1
F-measure	Mean	0.881533	0.943461	0.858099	0.86981	-	0.976459
	Std	3.38761 × 10^−16^	0	0.0256338	0.0270183	-	0.0337187
	Worst	0.881533	0.943461	0.79845	0.825811	-	0.854329
	Best	0.881533	0.943461	0.898216	0.937594	-	1
RI	Mean	0.986993	0.992146	0.983747	0.975294	-	0.996954
	Std	3.38761 × 10^−16^	4.51681 × 10^−16^	0.00267911	0.00821687	-	0.00554023
	Worst	0.986993	0.992146	0.977668	0.960883	-	0.971924
	Best	0.986993	0.992146	0.988004	0.993564	-	1

**Table 3:** The NMI values obtained by the experimental algorithms on the real-world networks with real partitions known.

Networks	NMI	FastNewman [16]	LconDanon [17]	GA-NET [6]	CMM [18]	Meme-Net [8]	EMCD-SOM
Karate Club	Mean	0.692467	0.530471	0.662719	1	0.759591	0.729539
	Std	2.25841e × 10^−16^	0	0.041038	0	0.12226	0.0916947
	Worst	0.692467	0.530471	0.593038	1	0.699488	0.6895798
	Best	0.692467	0.530471	0.707135	1	1	1
Football Club	Mean	0.697732	0.72976	0.36438	0.900688	0.877428	0.900987
	Std	1.1292 × 10^−16^	3.38761 × 10^−16^	0.0326597	0.00603723	0.0338035	0.0128863
	Worst	0.697732	0.72976	0.287833	0.896274	0.757927	0.858186
	Best	0.697732	0.72976	0.432277	0.914376	0.924195	0.91137
Political Book	Mean	0.530814	0.522288	0.407465	0.454128	0.46474	0.528597
	Std	4.51681 × 10^−16^	2.25841 × 10^−16^	0.0204818	3.38761 × 10^−16^	0.0283599	0.0190332
	Worst	0.530814	0.522288	0.361427	0.454128	0.425702	0.482507
	Best	0.530814	0.522288	0.449338	0.454128	0.522001	0.553662
Dolphins	Mean	0.5727	0.574277	0.431174	0.814113	0.52687	0.567711
	Std	1.1292 × 10^−16^	2.25841 × 10^−16^	0.0350064	1.1292 × 10^−16^	0.0510336	0.0432212
	Worst	0.5727	0.574277	0.363285	0.814113	0.396634	0.501266
	Best	0.5727	0.574277	0.523461	0.814113	0.612508	0.660154

**Table 4:** The F-measure values obtained by the experimental algorithms on real-world networks with real partitions known.

Networks	F-measure	FastNewman [16]	LconDanon [17]	GA-NET [6]	CMM [18]	Meme-Net [8]	EMCD-SOM
Karate Club	Mean	0.828011	0.758621	0.810516	0.812349	0.907227	0.89563
	Std	4.51681 × 10^−16^	3.38761 × 10^−16^	0.0345437	0.0292515	0.0471795	0.0353847
	Worst	0.828011	0.758621	0.761594	0.771371	0.884034	0.884034
	Best	0.828011	0.758621	0.846678	0.878937	1	1
Football Club	Mean	0.607997	0.624275	0.357385	0.888643	0.829276	0.881271
	Std	3.38761 × 10^−16^	4.51681 × 10^−16^	0.0259086	0.0102019	0.0593904	0.0222667
	Worst	0.607997	0.624275	0.304809	0.866702	0.654615	0.806481
	Best	0.607997	0.624275	0.415762	0.902567	0.914482	0.896491
Political Book	Mean	0.819664	0.792252	0.631611	0.778402	0.721159	0.810397
	Std	1.1292 × 10^−16^	2.25841 × 10^−16^	0.0476347	1.1292 × 10^−16^	0.0532029	0.0256946
	Worst	0.819664	0.792252	0.541227	0.778402	0.617422	0.736497
	Best	0.819664	0.792252	0.700829	0.778402	0.806321	0.834708
Dolphins	Mean	0.786624	0.70509	0.549487	0.968117	0.671548	0.721252
	Std	0	3.38761 × 10^−16^	0.056409	0	0.0584518	0.0520816
	Worst	0.786624	0.70509	0.444878	0.968117	0.567638	0.665973
	Best	0.786624	0.70509	0.753607	0.968117	0.778187	0.88149

**Table 5:** The Rand Index values obtained by the experimental algorithms on real-world networks with real partitions known.

Networks	RI	FastNewman [16]	LconDanon [17]	GA-NET [6]	CMM [18]	Meme-net [8]	EMCD-SOM
Karate Club	Mean	0.841355	0.707665	0.770291	0.762686	0.88164	0.866845
	Std	2.25841 × 10^−16^	2.25841 × 10^−16^	0.0276138	0.0295904	0.0601917	0.0451438
	Worst	0.841355	0.707665	0.730838	0.734403	0.85205	0.85205
	Best	0.841355	0.707665	0.802139	0.834225	1	1
Football Club	Mean	0.880702	0.887109	0.836476	0.971647	0.953755	0.973221
	Std	4.51681 × 10^−16^	5.64601 × 10^−16^	0.0252958	0.00177524	0.0241369	0.00652113
	Worst	0.880702	0.887109	0.762319	0.972387	0.886651	0.949352
	Best	0.880702	0.887109	0.88177	0.979863	0.984744	0.978032
Political Book	Mean	0.828205	0.804212	0.703199	0.759341	0.757045	0.820733
	Std	2.25841 × 10^−16^	1.1292 × 10^−16^	0.0192073	5.64601 × 10^−16^	0.034364	0.0203903
	Worst	0.828205	0.804212	0.6663	0.759341	0.707692	0.764103
	Best	0.828205	0.804212	0.730403	0.759341	0.817216	0.843223
Dolphins	Mean	0.713908	0.684294	0.570739	0.936542	0.645672	0.679129
	Std	3.38761 × 10^−16^	2.25841 × 10^−16^	0.0295801	0	0.0288785	0.0398455
	Worst	0.713908	0.684294	0.52935	0.936542	0.597039	0.640402
	Best	0.713908	0.684294	0.700159	0.936542	0.718139	0.814384
